# BIOLOGICAL RESOURCES FOR AGING RESEARCH AT THE NATIONAL INSITUTE ON AGING

**DOI:** 10.1093/geroni/igae098.2006

**Published:** 2024-12-31

**Authors:** Manuel Moro, Tiziana Cogliati, Jennifer Fox, Fei Wang

**Affiliations:** NIA-NIH, Bethesda, Maryland, United States; NIA-NIH, Bethesda, Maryland, United States; NIA-NIH, Bethesda, Maryland, United States; NIA-NIH, Bethesda, Maryland, United States

## Abstract

Research on aging, by its nature, requires the acquisition of data over an organism’s lifespan or at later stages in life. For animals with medium to long lifespans such as rodents and nonhuman primates (NHPs), time is a limiting factor for the acquisition of data and samples. One of the missions of the National Institute on Aging (NIA) is to maintain and manage biological resources needed to support aging research that would be otherwise less accessible or excessively costly or time consuming to the investigators. The resources made available to publicly funded investigators comprise: 1) live rodents (Aging Mouse and Rat Colonies); 2) cells from different species including human, NHP, rodent, and domestic companion animals (Aging Cell Repository); 3) tissues from aged rodents (Rodent Tissue Bank) and NHPs (NHP Tissue Bank); and 4) a database of NHP vital parameters (Primate Aging Database). The NIA also supports two multi-institutional programs investigating interventions with the potential to extend lifespan and delay disease/dysfunction in worms (Caenorhabditis Intervention Testing Program, CITP) and in mice (Interventions Testing Program, ITP). The NIA values feedback to continue to provide valuable resources that will help the field move forward.

